# The use of *Lactobacillus casei* DG® prevents symptomatic episodes and reduces the antibiotic use in patients affected by chronic bacterial prostatitis: results from a phase IV study

**DOI:** 10.1007/s00345-020-03580-7

**Published:** 2021-01-13

**Authors:** Tommaso Cai, Luca Gallelli, Erika Cione, Gianpaolo Perletti, Francesco Ciarleglio, Gianni Malossini, Giovanni De Pretis, Alessandro Palmieri, Vincenzo Mirone, Riccardo Bartoletti, Truls E. Bjerklund Johansen

**Affiliations:** 1 Department of Urology, Santa Chiara Regional Hospital, Trento, Italy; 2 Institute of Clinical Medicine, University of Oslo, Oslo, Norway; 3 Department of Health Sciences, University of Catanzaro, Catanzaro, Italy; 4 Clinical Pharmacology and Pharmacovigilance Unit, Mater Domini Hospital, Catanzaro, Italy; 5 Department of Pharmacy, Health and Nutritional Sciences, Department of Excellence 2018-2022, University of Calabria, 87036 Rende, CS Italy; 6 Department of Biotechnology and Life Sciences, Università degli Studi dell’Insubria, Varese, Italy; 7 Department of Human Structure and Repair, Faculty of Medicine and Medical Sciences, Ghent University, Ghent, Belgium; 8 Department of Surgery, Santa Chiara Regional Hospital, Trento, Italy; 9 Department of Gastroenterology, Santa Chiara Regional Hospital, Trento, Italy; 10 AOU Federico II – Urology Unit, University Federico II, Naples, Italy; 11 Department of Urology, University of Pisa, Pisa, Italy; 12 Department of Urology, Oslo University Hospital, Oslo, Norway; 13 Institute of Clinical Medicine, University of Aarhus, Aarhus, Denmark

## Abstract

**Purpose:**

To evaluate the efficacy of *Lactobacillus paracasei* CNCM I-1572 (L. casei DG®) in both prevention of symptomatic recurrences and improvement of quality of life in patients with chronic bacterial prostatitis (CBP).

**Methods:**

Patients with CBP attending a single Urological Institution were enrolled in this phase IV study. At enrollment, all patients were treated with antibiotics in agreement with EAU guidelines and then were treated with L. casei DG® (2 capsules/day for 3 months). Clinical and microbiological analyses were carried out before (enrollment, T0) and 6 months (T2) after the treatment. Both safety and adherence to the treatment were evaluated 3 months (T1) after the enrollment. NIH Chronic Prostatitis Symptom Index (CPSI), International Prostate Symptom Score (IPSS) and Quality of Well-Being (QoL) questionnaires were used. The outcome measures were the rate of symptomatic recurrence, changes in questionnaire symptom scores and the reduction of antibiotic use.

**Results:**

Eighty-four patients were included. At T2, 61 patients (72.6%) reported a clinical improvement of symptoms with a return to their clinical status before symptoms. A time dependent improvement in clinical symptoms with significant changes in NIH-CPSI, IPSS and QoL (mean difference T2 vs T0: 16.5 ± 3.58; − 11.0 ± 4.32; + 0.3 ± 0.09; *p* < 0.001), was reported. We recorded that L. casei DG® treatment induced a statistically significant decrease in both (*p* < 0.001) symptomatic recurrence [1.9/3 months vs 0.5/3 months] and antibiotic use [− 7938 UDD]. No clinically relevant adverse effects were reported.

**Conclusions:**

L. casei DG® prevents symptomatic recurrences and improves the quality of life in patients with CBP, reducing the antibiotic use.

## Introduction

Even though if the prevalence of chronic bacterial prostatitis (CBP), category II according to the National Institutes of Health (NIH) classification, ranges in Europe between 7 and 14% of all cases with prostatitis [1], the impact on patient’s quality of life is high [2, 3]. A antibiogram-driven long-term treatment with fluoroquinolones represents the gold standard therapy of CBP, but nowadays the need to improve the adherence to antibiotic stewardship programs forced us to re-think the approach to CBP. Several authors reported that the pathogenesis of CP seems related to the presence of bacterila biofilm [2] while clinical symptoms with the prostate inflammation mediated by several cytokines, e.g., Interleukin-8 [3–5]. On the other hand, other authors suggested that probiotics, in particular *Lacobacillus* strains, could modulate the inflammatory pathway regulating the bowel inflammatory status [6, 7], suggesting a role in prostatic diseases, too [8]. In agreement with this, Vicari et al., documented that probiotics play a role in the management of patients affected by CBP and irritable bowel syndrome but at the best of our knowledge, no study addressed the therapeutic role of Lactobacilli in the management of CBP patients evaluating its role in the decrease of antibiotic use [8]. Therefore, here we evaluated the efficacy of *Lactobacillus paracasei* CNCM I-1572 (L. casei DG®) in both the prevention of symptomatic recurrences and the improvement of the quality of life in CBP patients.

## Patients and methods

### Study design and participants

We performed a clinical, single center, phase IV study on CBP patients from January 2019 up to December 2019. This study was approved by the local Ethic Committee (approval protocol number 258, 2019) and its was conducted in compliance with the Institutional Review Board/Human Subjects Research Committee requirements and with the Declaration of Helsinki and with the Guidelines for Good Clinical Practice criteria. Before the beginning of the study, the enrolled patients or legal guardians signed the informed consent.

### Population inclusion criteria

In agreement with our previous studies [9], we enclosed men > 18 year and < 45 year, with symptoms related to CBP for at least 3 months and a positive Meares–Stamey 4-glass test with first voided urine, midstream urine, expressed prostatic secretion and a post-prostatic massage urine culture, which had to be ≥ 10 [3] colony forming units (CFU)/mL of uropathogens. Patients who had recently (< 4 weeks) undergone oral or parental treatment or who were currently using prophylactic antibiotic drugs were excluded. All patients with positive tests for atypical or sexually transmitted pathogens, such as *Chlamydia trachomatis*, *Ureaplasmaurealiticum*, or *Neisseria gonorrhoeae* were also excluded. To obtain a homogenous group to analyze the following bacteria were considered as uropathogens, in agreement with Trinchieri: enteric Gram-negative rods; enterococci, Staphylococcus saprophyticus; and group B streptococci [10]. Finally, all patients with clinically significant intestinal disease were excluded, to obtain results on efficacy of probiotic therapy on chronic bacterial prostatitis. Patients who did not sign the informed consent were also excluded.

### Experimental protocol

**Fig. 1:**
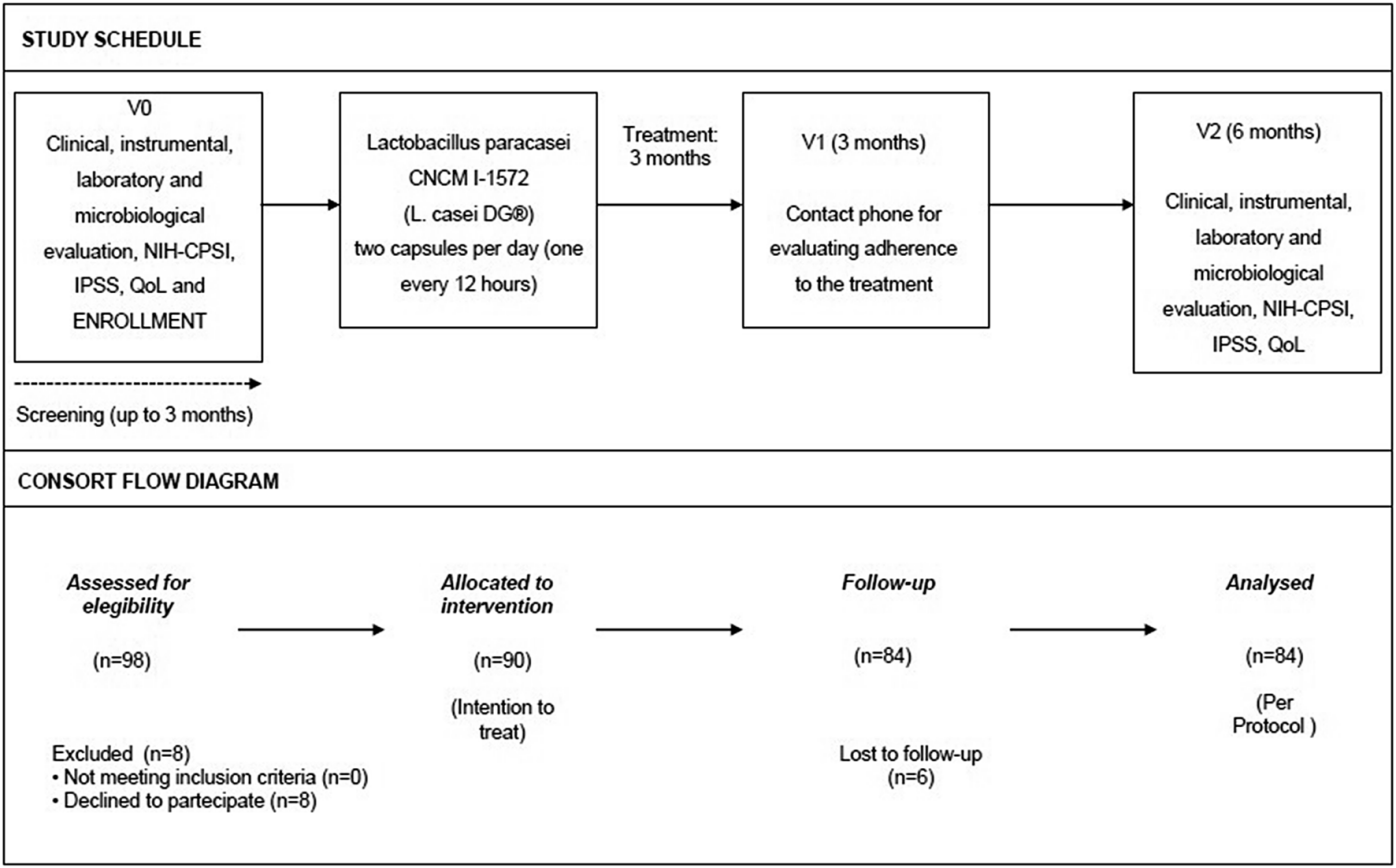
Study schedule and CONSORT flow diagram

At the enrolment, all patients were treated with antibacterial agents in agreement with European Association of Urology (EAU) guidelines [11], to obtain infection free status at baseline (T0) and then they received a treatment with L. casei DG®1 capsule/12 h for 3 months. At the first follow-up time point (3 months, T1), all patients were telephonically contacted to evaluate both the compliance and the safety of the treatment. Clinical and microbiological analyses were carried out at the enrolment (T0) and 6 months after the discontinuation of L. casei DG® (T2 time point). In these periods, the patients were asked to fill out the NIH Chronic Prostatitis Symptom Index (CPSI), International Prostatic Symptom Score (IPSS) and Quality of Well-Being (QoL) questionnaires. All patients underwent urologic visit and microbiological evaluation in presence of clinical recurrence, also. The study schedule and study flow chart are reported in Fig. 1.

### End-points

The primary efficacy end-point of this trial was the statistically significant improvement (*p* < 0.05) of clinical symptoms at T2 vs T0. The secondary end-point of efficacy was the statistically significant decrease (*p* < 0.05) in the use of antimicrobial drugs. The reduction of antibiotic use was calculated under the following criteria: the number of Used Daily Dose (UDD) of antibiotics at T0 was compared respect to T2 [UDDT2 –UDDT0; results < 0 the decrease of antibiotic use is relevant] in agreement with the paper of Monnet et al. [12] that documented a strong correlation between the Defined DD of antibiotic and the antimicrobial prescription. All patients enrolled in this study have been previously included in a prospective dedicated data-base (Advanced PROSTATitis DataBase, Microsoft Access format) [13]. The primary safety end-point was the presence/absence of significant differences in the rate of adverse drug effects between T2 and T0. In agreement with our previous paper, the Naranjo probability test was used to evaluate the correlation between adverse drug reaction and treatment [14].

### Clinical and microbiological definitions

Clinical efficacy was considered as being asymptomatic for at least 2 weeks. Clinical failure was defined as the persistence of clinical symptoms after treatment or the suspension of therapy for significant reported adverse effects. In addition, spontaneously reported adverse events or those noted by the investigator were recorded during the whole study period. The Meares–Stamey test was carried-out only in patients with symptomatic recurrence. All positive patients to Meares–Stamey test for uropathogens were treated with an alternative antibiotic depending on the organism and its susceptibility profile. Microbiological culture was carried out in accordance with the methods described in our previous papers [1, 2, 9, 13]. In brief, bacteria isolated from all samples were cultivated aerobically in Columbia blood agar (BioMerieux, Italy) and in a 10% CO2 atmosphere in Columbia CNA agar (BioMerieux; BD, Italy). They were identified and characterized biochemically using the species identification cards of the Vitek II semi-automated System for Microbiology-BioMerieux; antibiotic chemosensitivity has been carried-out using Vitek II semi-automated System for Microbiology (BioMerieux) [6]. Positive urine cultures had colony counts > 10 [5] UFC/mL [1, 2, 9, 13].

### Questionnaires and urological examinations

The validated Italian versions of the NIH Chronic Prostatitis Symptom Index (NIH-CPSI) [15] and the International Prostate Symptom Score (IPSS) [16] were administered to each patient and self-completed. The questionnaire was administered at the patient’s arrival at the Centre and the results collected in the dedicated database. Moreover, patient quality of life was measured using an Italian version of the Quality of Well-Being, a validated, multi-attribute health scale [17]. In accordance with the study by Nickel et al., prostatitis-like symptoms were considered significant at a pain score of ≥ 4. The NIH-CPSI was also used in determining clinical therapy efficacy [18].

### Composition and formulation of probiotics used in this trial

The probiotic preparation consisted of a gelatine capsule containing at least 24 billion viable cells of the bacterial strain L. casei DG® (*Lactobacillus paracasei* CNCM I-1572), Enterolactis® plus (SOFAR S.p.A., Trezzano Rosa, Milan, Italy) deposited at Institute Pasteur of Paris with number I1572. Probiotic capsules were delivered in aluminium boxes sealed with a plastic cap containing desiccant salts.

### Statistical inference

This study has been planned as prospective phase IV study. To obtain clinically significant results to analyze, sample size calculation was based on the following assumptions: difference in terms of recurrence between enrollment (at the end of antibiotic treatment period) and follow-up visit (6 months): −1/3 months ± 1; α error level, 0.05 two-sided; statistical power, 80%; anticipated effect size, Cohen’s d = 0.5. The sample size calculation yielded 80 individuals. Considering a drop-out rate of at least 10%, the final sample size has been set to 90 patients. At baseline, the independent sample two-tailed *t* test was used to compare variables. For categorical parameters, chi-square test was applied. Changes from baseline to end of therapy were analyzed using ranked one-way analysis of variance (ANOVA) with a term for treatment group. The Shapiro-Wilks’s test for normality has been used. Data were reported as means ± standard deviation (SD). For all statistical comparisons, significance was considered as *p* < 0.05. All reported *p* values are two-sided. All statistical analyses were performed using SPSS 11.0 for Apple-Macintosh (SPSS, Inc., Chicago, Illinois). No placebo run-in period was considered necessary for the treatment of those patients showing Meares-Stamey test positivity. All data recorded in this study, i.e., anamnestic, clinical, and laboratory data, containing sensitive information were deidentified to ensure analysis of anonymous data only. This process was performed by non-medical staff using dedicated software.

## Results

Ninety-eight patients were screened and admitted to this study. Eight patients (8.2%) refused to be enrolled, while 90 patients were enrolled (Intention To Treat group—ITT) (91.8%). 6 patients were lost (6.7%) to the follow-up and 84 completed the study (Per Protocol group—PP).

### Baseline characteristics (T0)

Anamnestic, clinical, microbiological and questionnaires data at the enrolled patients are reported in Table 1.

**Table 1:** Demographic, clinical, laboratory and microbiological patient’s data at the enrolment time

	Total or mean (SD^*^ or %)
Patients	84
*Age*	36.1 ± 6.8
*Educational qualification*	
Primary School	–
High School	63 (74.9)
University	21 (25.1)
*Sexual behaviour*	
1 partner	79 (94.1)
> 1 partners	5 (3.9)
*Contraceptive use*	
Condom	43 (51.2)
Coitus interruptus	41 (48.8)
*Start of CBP *^*#*^* history (months)*	23.9 ± 5.9
*Symptoms Score at baseline*	
NIH-CPSI^§^	20.2 ± 2.3
IPSS^°^	18.4 ± 3.4
QoL^‡^	0.57 ± 0.1
*Clinical presentation*	
Burning	52 (62.5)
Tenesmus	16 (19.1)
Painful micturition	69 (82.1)
Dysuria + Frequency	38 (45.2)
Urgency	22 (26.2)
*Previous treatments (*> *4 weeks before enrolment)*	
Alpha-blockers	8 (0.9)
Antibiotics	84 (100)
Anti-inflammatory drugs	24 (28.5)
Phytotherapy	30 (35.7)
Antibiotics + Phytotherapy	55 (65.4)
Antibiotics + Anti-inflammatory	23 (27.8)
*Microbiological findings*	
Positive Meares–Stamey test	84 (100)
*Escherichia coli*	47 (55.9)
*Enterococcus faecalis*	25 (29.7)
Other uropathogens	12 (14.4)
Klebsiella spp.	6 (50.0)
Serratia spp.	4 (33.3)
Entrobacter spp.	2 (16.7)
No growth	0 (–)

The table shows all baseline characteristics and clinical parameters at visit 0. SD^*^ = Standard Deviation; CBP^#^ = Chronic Bacterial Prostatitis; NIH-CPSI^§^ = NIH Chronic Prostatitis Symptom Index; IPSS° = International Prostate Symptoms Score; QoL^‡^ = Quality of Well-Being questionnaires

### Adherence and adverse events (T1)

All patients correctly took all doses without any discontinuation, showing compliance with the study protocol of 100%. Two patients had mild adverse effects (mild dyspepsia) that did not require treatment suspension. No severe adverse effects have been reported.

### Follow-up 6 months (T2)

**Fig. 2:**
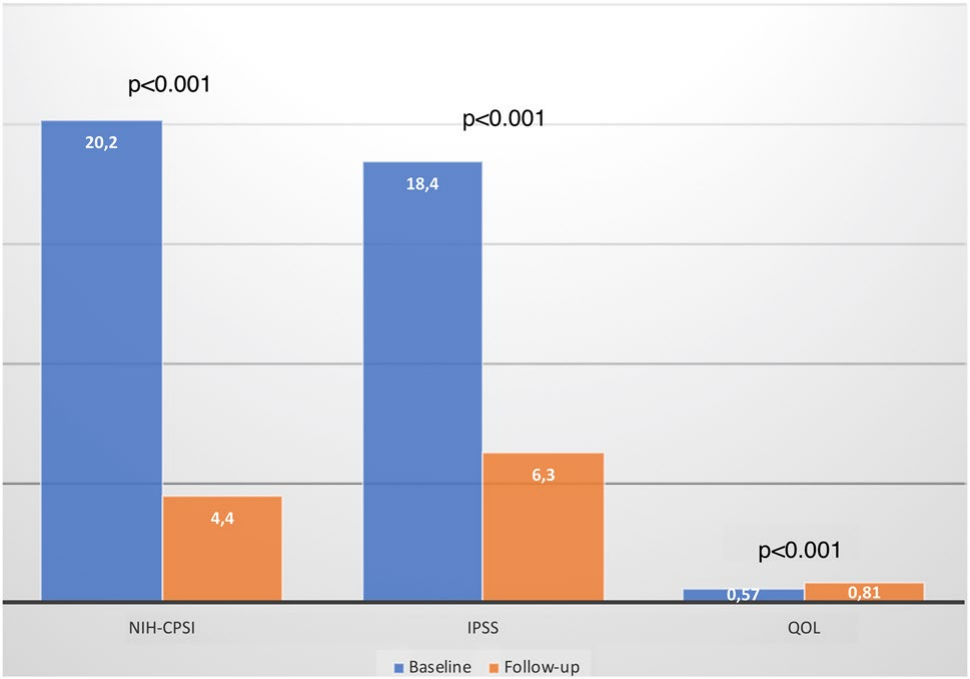
NIH-CPSI, IPSS and QoL scores at baseline and follow-up evaluations

At the end of follow-up period, 61 patients (72.6%) reported a significant clinical improvement (first safety end-point). A statistically significant reduction of symptomatic recurrence rate has been reported between T2 and T0 [1.9/3 months vs 0.5/3 months (*p* < 0.001)]. At T2, we documented significant changes in the score of NIH-CPSI, IPSS and QoL compared to T0 (mean difference: -16.5 ± 3.58; -11.0 ± 4.32; + 0.3 ± 0.09; *p* < 0.001; *p* < 0.001; *p* < 0.001, respectively) (Fig. 2). Table 2 reports the mean change differences from T2 to T0. The UDD at T0 was 9,525.6, while at T2 was 1,587.6 with a statistically significant difference in terms of antibiotics used [− 7938 (*p* < 0.001)] (secondary efficacy end-point). The Table 3 shows all microbiological findings in patients with symptomatic recurrence and mean antimicrobial resistance profiles of all bacterial isolates at enrolment and follow-up visit.

**Table 2:** Questionnaire results at the 6 months follow-up visit

	Pre-treatment	Post-treatment	*p*
	Mean (SD^*^)	Mean (SD^*^)	
NIH-CPSI^#^			
*pre-treatment*	20.2 ± 2.3	4.4 ± 2.1	< 0.001
Mean difference	− 16.5 ± 3.58		
IPSS^°^			
*pre-treatment*	18.4 ± 3.4	6.3 ± 2.4	< 0.001
Mean difference	− 11.0 ± 4.3		
QoL^‡^			
*pre-treatment*	0.57 ± 0.1	0.81 ± 0.1	< 0.001
Mean difference	+ 0.3 ± 0.09		

The table shows all questionnaire results at the follow-up visit. SD^*^ = Standard Deviation; NIH-CPSI^#^ = NIH Chronic Prostatitis Symptom Index; IPSS^°^ = International Prostate Symptoms score; QoL^‡^ = Quality of Well-Being questionnaires

**Table 3:** Microbiological findings in patients with symptomatic recurrence and mean antimicrobial resistance profiles of all bacterial isolates at enrolment and follow-up visit

Patients with symptomatic recurrence at T2 time point	23 (27.4%)								
	Antimicrobial agents tested								
	Resistance (%)								
	GM	CPFX	CTX	LVFX	IPM	PIPC/TAZ	SMX/TMP	ABPC	VCM
*Isolated bacteria at T0 (baseline)*									
*Escherichia coli* (14)	7.1	57.1	0	57.1	0	0	21.4	42.8	–
*Enterococcus faecalis* (9)	66.6	55.5	22.2	33.3	11.1	11.1	33.3	33.3	0

	Antimicrobial agents tested									
	Resistance (%)									
	GM	CPFX	CTX	LVFX		IPM	PIPC/TAZ	SMX/TMP	ABPC	VCM
Isolated bacteria at T2 time point (6 months)										
*Escherichia coli* (10)	30	70	10	70		0	0	30	40	–
*Enterococcus faecalis* (12)	58.3	33.3	8.3	33.3		0	0	25	16.6	0
*Klebsiella oxytoca* (1)	0	0	0	0		0	0	0	–	–

The table shows all microbiological findings in patients with symptomatic recurrence and mean antimicrobial resistance profiles of all bacterial isolates at T0 and T2 time point. GM = gentamicin; CPFX = ciprofloxacin; CTX = cefotaxime; LVFX = levofloxacin; IPM = imipenem; PIPC/TAZ = piperacillin/tazobactam; SMX/TMp = sulfamethoxazole-trimethoprim; ABPC = ampicillin; VCM = vancomycin

## Discussion

In this study, we demonstrated for the first time, that in patients with CBP, the treatment with L. casei DG® prevents the symptomatic recurrences, improving the quality of life and reducing the antibiotic use. Moreover, we demonstrated full treatment compliance, as no study discontinuations were recorded. The high compliance is related to the low frequency of adverse events and the efficacy of the treatment in terms of quality of life improvement.

### Results in the context of previous studies

CBP continues to pose a treatment challenge for all urologists and for these reasons, a lot of non-standardized treatment schedule, sometimes in off-label way, were offered to the patients. Promising results are emerging from studies focusing on the microbiota of patients. Recent data acquisition about the role of the microbiome in healthy humans, allowed us to understand that there exists interplay and symbiotic relationships between our bodies and the microorganisms colonizing the gastro-intestinal system [19]. Recent studies indicated that the microbiome can influence prostate inflammation in relation to benign prostate conditions such as prostatitis/chronic pelvic pain syndrome and benign prostatic hyperplasia, as well as in prostate cancer [20]. Starting from these considerations, the reduction of antibiotics and the maintenance of normal gut homeostasis should be considered also in the management of bacterial prostatitis. Vicari and co-workers enrolled a total of 106 infertile male patients affected by CBP and irritable bowel syndrome [8]. All patients underwent rifaximin treatment in combination with probiotics containing multiple bacterial strains and compared the clinical results with a no treatment control group [8]. They concluded that a long-term treatment with rifaximin and probiotics is effective in lowering the progression of prostatitis into more complicated forms of male accessory gland infections [8]. Unlike Vicari’s study, in our study, we enrolled patients without any intestinal disease and we treated them with probiotics only. On the other hand, another Italian group, demonstrated that a 6 months treatment with probiotics plus *Vaccinium Macracarpon* and *Lyciumbarbarum L.*, reduces the number of symptomatic episodes and improves the quality of life [21]. The efficacy of probiotics in the management of CBP could be related to the immune modulation of the bowel epithelium with the suppression of the low-grade inflammation [8, 22] and with the decrease of uropathogens spreading through the bowel mucosa. Here, we selected a specific probiotic strain, L. casei DG®, that has been demonstrated to modulate the intestinal microbial ecosystems of healthy adults and patients affected by inflammatory bowel disease, and to influence host immune response via its unique polysaccharide capsule [23–25]. L. casei DG® has also been demonstrated as therapeutic potential for several dysfunctions and pathological conditions such as increasing the efficacy of antibiotic eradication therapy against H. pylori [26].

### Strengths and limitations of this study

Even if our results are encouraging, this study shows several limitations to consider. Firstly, the number of enrolled patients. Even if the number need to treat is correctly calculated is very important to highlight that the efficacy and safety of probiotics should be evaluated with a long-term follow-up, to discover delayed adverse side effects. However, taking into consideration the studies evaluating the long-term therapy with L. casei DG® in other medical setting, we could consider this treatment safe also in CBP [27]. The use of a short-term antibiotic treatment period should not be considered a limitation of the study. In accordance with Bjerklund Johansen et al., who stated that the minimum duration of antibiotic treatment should be 2–4 weeks, we chosen this 14-day treatment course [27]. Finally, the lack of a control group should be considered a limitation of the study. Moreover, the role of a possible placebo effect should be considered as a factor influencing the patients’ outcome. However, in this Phase IV study we considered the following end-points: treatment efficacy defined as improvement of clinical symptoms at T2 vs T0, and a decrease in the use of antimicrobial drugs at T2 vs T0. In this sense, even though we did not consider a control group in our study design, the efficacy of L. casei DG® has been demonstrated by performing a paired comparison between pre- (T0) and post-treatment (T2) outcomes, in line with Thomas Jaeger [28]. Moreover, comparison of the results of this study with a cohort of historical controls, assessed 6 months after the end of antibacterial treatment suggests that L. casei DG® may have concurred to a sustained reduction of NIH-CPSI and IPSS scores (19.8 ± 1.9 and 17.9 ± 3.3 in the historical cohort), and may have had an influence in decreasing the recurrence rate (1.7/3 months) and the antibiotic usage[9,13]. However, future studies with randomized and blinded design are needed to confirm these results.

### Implications for clinical practice

The results of this study should be read in the light of the continuing increase of resistant bacterial strains and the necessity to find new strategies to reduce the use of antibiotics in CBP. Our results suggest that L. casei DG® reduces both the symptomatic recurrence and the use of antibiotics. Therefore, the use of L. casei DG® after antibiotic treatment represents a valid tool to improve the antibiotic stewardship in urological setting. In an economic perspective, our findings suggest a reduction in direct costs related to the reduction of antibiotic daily dose and indirect costs related to the patients’ well-being (less lost working days, less stress and anxiety), even if we did not revaluate this economic outcome.

## Conclusions

In patients with CBP, L. casei DG® is able to prevent symptomatic recurrences, improving the quality of life and reducing the antibiotic use. Future larger clinical trials with a randomized and blinded design are needed to confirm these results especially in terms of economic perspectives.
